# Automated Ergonomic Risk Assessment of Wheelchair Users During Cabinet Interaction Using Vision-Based 3D Pose Estimation

**DOI:** 10.3390/s26092893

**Published:** 2026-05-05

**Authors:** Yilin Xu, Ziqian Yang, Tao Sun, Jiachuan Ning

**Affiliations:** 1 College of Furnishings and Industrial Design, Nanjing Forestry University, Nanjing 210037, China; 2 Nanjing Institute of Agricultural Mechanization, Ministry of Agriculture and Rural Affairs, Nanjing 210049, China; 3 Qingdao Grace Chain Software Ltd., Qingdao 266071, China

**Keywords:** 3D pose estimation, ergonomic risk assessment, wheelchair users, monocular vision, fuzzy RULA, human motion analysis

## Abstract

Advanced sensor signal analysis is increasingly important for intelligent health management in human-centered environments, where continuous perception and real-time interpretation of motion-related signals are essential for safe and adaptive assistance. In this study, we propose a vision-based sensor signal analysis framework for automated ergonomic risk assessment of wheelchair users during cabinet interaction. The proposed framework integrates YOLOv11 for human detection, MHFormer for monocular 3D pose reconstruction, and a fuzzy logic-enhanced RULA model for continuous ergonomic risk quantification from video-derived motion signals. To support model development and evaluation, we constructed a dedicated wheelchair cabinet-operation dataset comprising 30 participants, including 14 experienced wheelchair users and 16 trained simulation participants, across five representative cabinet-operation scenarios. The raw dataset contained approximately 5 h of RGB video and about 150,000 original frames. To reduce redundancy caused by highly similar consecutive frames and to mitigate overfitting risk, representative frames were sampled from the continuous video sequences, resulting in 10,000 images for annotation and model development. Based on the proposed framework, raw visual sensor signals are transformed into temporally continuous kinematic representations and ergonomic risk scores, enabling non-contact and real-time health-state interpretation in assistive living environments. The proposed method achieved an average joint-angle estimation RMSE of 7.5°, representing an approximately 60% reduction compared with a Kinect v2-based motion capture baseline (18.6°), which is widely used for low-cost ergonomic evaluation. In benchmark evaluation, the proposed method achieved 84% risk-classification accuracy with a Cohen’s kappa of 0.66, outperforming representative baseline approaches. The results further indicated that low revolving-door and low-drawer operations were associated with higher and more sustained ergonomic risk exposure than sliding-door interaction. These findings demonstrate that vision-based sensor signal analysis can provide an effective solution for intelligent health management, ergonomic monitoring, and perception-driven assessment in accessible and assistive autonomous living systems.

## 1. Introduction

The global wheelchair user population continues to expand significantly, encompassing individuals with lower limb disabilities, elderly persons with mobility limitations, and patients undergoing rehabilitation. According to official statistics reported by the China Disabled Persons’ Federation, China had approximately 85 million persons with disabilities as of 2020, highlighting the large population potentially affected by accessibility-related challenges [1]. This demographic transformation, coupled with rapid population aging worldwide, has intensified the urgent need for accessible home environments that effectively accommodate wheelchair users’ specific functional requirements.

Wheelchair users encounter distinctive challenges when interacting with standard household furniture, particularly cabinet systems designed for standing users. Unlike their ambulatory counterparts, wheelchair users must perform cabinet operations from a seated position with inherently limited mobility, frequently necessitating compensatory upper limb movements including excessive shoulder abduction, wrist hyperflexion, and trunk rotation to overcome spatial and mechanical constraints. The long-term repetition and heavy load on the body can easily lead to work-related musculoskeletal injury. Among them, the impact on shoulder structure is particularly obvious, because it not only undertakes the task of wheelchair pushing, but also completes various movements in daily life. Previous studies have reported that the prevalence of shoulder pain among manual wheelchair users ranges from 36% to 71% [2]. These findings indicate that reducing shoulder load is a critical issue in the long-term use of wheelchairs.

Current furniture design standards use body measurement data from people who stand. These standards include heights for worktops and cabinets. However, they largely ignore the specific reach ranges of users who are seated [3]. When wheelchair users interact with this incompatible furniture, they lack sufficient support for their lower limbs. This forces them to use compensating body postures to complete tasks. Research shows that wheelchair users must reach high or deep into storage. To do this, they often move their arms far out to the side. They also extend their wrists backward too much. Sometimes they twist or bend their upper body a great deal [4]. These positions are not neutral. They reduce efficiency during tasks. They also place much higher biomechanical stress on the shoulder and wrist joints. This stress can narrow the space under a part of the shoulder bone. As a result, the risk of injuring the rotator cuff tendons increases significantly [5].

Wheelchair users rely heavily on their upper limbs in daily life. They use them for propulsion and for handling objects. This use is repetitive and places high demands on the body. As a result, the incidence of work-related musculoskeletal disorders (WMSDs) is very high. Multiple epidemiological studies report an extremely high prevalence of shoulder pain among manual wheelchair users. For those who use wheelchairs for a long time, the incidence of shoulder pain will further increase [6,7]. Shoulder pain is not only physically uncomfortable, but also the main reason for the decline of independent living ability of wheelchair users, which seriously affects their quality of life [7]. The so-called unreasonable environment, such as the design of kitchen or storage space, does not conform to ergonomics, which increases the burden of upper limbs [5,8]. The most common types of shoulder injury in the population are rotator cuff impingement syndrome and rotator cuff tear, which is directly related to their frequent high-intensity upper limb activities in unreasonable environments. Such environments include poorly designed kitchens or storage spaces.

Rapid upper limb assessment (RULA) and rapid entire body assessment, (REBA) are the most widely used tools, and are regarded as the gold standard for assessing the risk of work-related musculoskeletal disorders (WMSDs). Its limitation is that it heavily relies on manual observation, which limits its application in large-scale or dynamic scenes. During the evaluation process, experts are required to manually measure or visually measure the joint angle. This method is not only time-consuming and laborious, but also has significant differences among evaluators [9]. Research shows that it is difficult for different evaluators to give consistent scores for the same posture [10], and the scores are easily affected by subjective factors such as the observer’s perspective and experience level [10]. The traditional method usually adopts snapshot evaluation, and only selects the most extreme single moment in the task process for scoring. This discontinuous sampling method cannot capture the dynamic changes of biomechanical load in the whole operation cycle, resulting in the underestimated cumulative injury risk [11]. However, these limitations make it difficult to perform continuous and objective ergonomic assessment in dynamic real-world tasks.

In addition to the limitations discussed above, the traditional RULA method also suffers from issues related to its discrete scoring mechanism. The traditional RULA method uses a discrete grading system, which simplifies the calculation process, but will lose a lot of continuous information with clinical significance. In practice, a slight posture adjustment may lead to a sudden jump in the risk score. This mutation cannot accurately reflect the smooth accumulation process of musculoskeletal stress [12]. A number of comparative studies have shown that the discrete scoring system is difficult to quantify the effect of subtle posture improvement, nor can it provide high-resolution risk feedback, and is not accurate enough in scenes requiring fine design intervention [13]. In order to overcome these limitations, recent studies have increasingly tended to introduce fuzzy logic or continuous algorithm into risk assessment. This transformation aims to shift from qualitative classification to quantitative, and realize continuous measurement [14].

To address the limitations of traditional ergonomic assessment methods, vision-based approaches have been increasingly explored in recent years. There are limitations in the application of traditional monocular vision system in complex environment. In order to solve this problem, the current ergonomics evaluation system increasingly adopts the combined technology path of detection before reconstruction. The research shows that the single-stage object detection algorithm like YOLO has become the most mainstream front-end processing tool in human detection tasks because of its excellent real-time processing ability and recognition stability. The recognition accuracy of the new generation of YOLO model represented by YOLOv8 or v11 is extremely high, and it can accurately lock the target even when the background is disordered or the human body is dynamically occluded, so as to provide a stable image input area for subsequent pose analysis [15]. However, only the detection of two-dimensional images cannot provide the depth information necessary for biomechanical analysis. For this reason, the researchers introduced the vision converter technology to solve the key problem of mapping from two-dimensional image to three-dimensional space. In particular, the introduction of multi hypothesis converter (MHFormer) effectively solves the core bottleneck of monocular 3D pose estimation, depth ambiguity and self occlusion. This method does not directly output a unique attitude judgment, but generates a number of reasonable attitude assumptions by learning a variety of possible spatio-temporal attitude representations, and then fuses them by weighting, so as to obtain more reliable estimation results. When dealing with the limb occlusion problem of wheelchair users caused by equipment structure, this method shows stronger robustness than traditional graph convolution networks (GCNs) [16].

Although vision-based pose estimation improves motion capture accuracy, it does not directly provide ergonomic risk evaluation. The traditional rapid upper limb assessment (RULA) uses a hierarchical discrete scoring standard, which is prone to sudden jump of scoring results near the critical point. To solve this problem, the introduction of fuzzy logic has become a key strategy to improve the accuracy of evaluation. The traditional RULA uses a clear ‘binary’ boundary standard, which often hides the real risk near the critical threshold. In order to solve this problem, the trapezoidal membership function with smooth transition characteristics has been proposed to replace the traditional rigid dichotomy boundary. This method maps joint angles into continuous degree of membership values [14]. This fuzzification process allows the assessment system to capture the smooth transition from ‘slight discomfort’ to ‘severe risk’. For instance, when a joint angle is in a critical range, the system no longer outputs a single risk score. Instead, a fuzzy inference engine calculates a weighted risk index. Research confirms that using the centroid method for defuzzification can output a continuous risk score. This score is more sensitive than the traditional RULA score. Therefore, it can more accurately reflect the biomechanical pressure linearly accumulated with posture deterioration [17]. Therefore, continuous risk modeling based on fuzzy logic provides a more suitable framework for ergonomic assessment in dynamic interaction scenarios.

In the proposed fuzzy RULA framework, joint kinematic variables, including shoulder abduction, elbow flexion, and trunk inclination, are first mapped into linguistic variables (e.g., low, medium, high, and very high risk) using trapezoidal membership functions. Based on these fuzzy representations, a rule-based inference mechanism is constructed to evaluate ergonomic risk under different posture combinations. The fuzzy rules are derived from standard RULA scoring principles and extended to support continuous risk assessment. For instance, when the shoulder abduction angle exceeds the neutral range while the elbow joint is in a non-neutral configuration, the corresponding ergonomic risk is classified as high. In contrast, when all joint angles remain within their respective neutral ranges, the risk level is considered low. Intermediate conditions involving moderate deviations across multiple joints are mapped to medium risk levels. By integrating multi-joint posture information, the fuzzy inference system enables a more flexible and continuous representation of ergonomic risk compared to traditional discrete RULA scoring. Representative fuzzy inference rules used in this study are summarized in Table 1.

The Kinect V2 sensor developed by Microsoft has been widely adopted as a standard low-cost device for ergonomic evaluation and motion capture. However, previous studies have reported notable limitations in its ability to accurately capture fine-grained joint movements. In particular, its performance degrades under conditions involving occlusion (e.g., interference from wheelchair structures) and complex seated postures. Existing literature indicates that the root mean square error (RMSE) of Kinect V2 in estimating upper-limb joint angles typically ranges from 8.5° to 13.0°. Under more challenging scenarios, such as partial occlusion or constrained movement configurations, the estimation error may further increase, thereby affecting the reliability of ergonomic assessment results [18,19]. In contrast, recent advances in deep learning-based human pose estimation, particularly Transformer-based models, have demonstrated substantial performance improvements. Models such as PoseRx and MHFormer leverage self-attention mechanisms to capture spatio-temporal dependencies in video sequences, enabling more robust estimation of occluded joints and reducing the mean absolute error (MAE) to approximately 5.4° [15,20]. These developments provide a strong foundation for high-precision automated ergonomic assessment and support the objective of this study to achieve improved joint-angle estimation accuracy (RMSE: 7.5°). Despite these advancements, limitations remain in applying existing methods to specific daily interaction scenarios.

A number of studies in the field of biomechanics have pointed out that the long-term excessive or unnatural posture of the upper limb joints is an important early warning indicator for the occurrence of musculoskeletal injury in wheelchair users. A large number of clinical studies have further confirmed that there is a significant positive correlation between this extreme posture and injury risk. Research data show that there is a close correlation between the movement mode of some specific joints and the shoulder pain score (i.e., WUSPI index) of wheelchair users [18]. These specific joint movements mainly include abduction of shoulder joint and flexion and extension of elbow joint. Among them, the angle change of elbow joint is considered to be a key parameter, because it cannot only reflect the efficiency of wheelchair propulsion, but also reflect the load borne by the joint. Studies have shown that, in order to reduce the risk of subacromial impingement syndrome, wheelchair users should try to maintain the elbow angle within the ideal range of 100 degrees to 120 degrees when performing daily operations [19]. If the joint movement beyond this range is repeated for a long time, the shoulder will bear additional load due to the compensation mechanism. In addition, the maximum distance of the arm extending forward has also been proved to be an important variable, which is closely related to the user’s torso stability and fall risk [4]. When the arm is stretched forward excessively, the center of gravity of the human body is easy to exceed the support range of the seat, thus causing high-risk compensatory contraction of muscles to maintain body balance.

In the study of barrier free design, the choice of subjects is crucial to the effectiveness of the results. Through the comparative analysis, it can be found that different groups of people have significantly different movement styles when performing the same action. This difference is mainly reflected in the operation comparison between professionals who use wheelchairs for a long time and healthy simulators. Professional wheelchair users usually adopt more efficient implementation methods, such as arc drawing semi-circular wheel pushing action. While maintaining the pushing speed, the muscle coordination mode is also more coordinated and optimized, but this efficient pushing mode sometimes brings greater load to the joints in an instant [20]. In contrast, sound simulators often have to rely more on arm strength to make up for the lack of effective control over the trunk core muscles, so as to form compensatory actions. This essential difference shows that there is a fundamental difference between the mechanical characteristics of the simulator and the real wheelchair user in the process of movement [21]. This finding emphasizes a key principle in the research design, that is, when evaluating the possible risks in the interaction between wheelchair users and furniture and other facilities, the data of real users must be collected for verification. If only relying on the performance of simulators, it is likely to ignore the unique physiological adaptation mechanism formed by long-term disability, resulting in deviation in the risk assessment [22].

Because of its excellent ability of target detection and real-time pose estimation, YOLOv11 has been widely and deeply applied in risk assessment and pose monitoring in the field of ergonomics. In terms of medical ergonomics, researchers have developed real-time working posture analysis systems for surgeons and dental practitioners using YOLOv11 [23,24]. These systems can calculate joint angles in real time, classify dangerous postures and provide automated corrective feedback by accurately extracting key bone points and combining classic ergonomic models such as RULA (rapid upper limb assessment) and Reba (rapid whole body assessment), so as to effectively reduce the risk of musculoskeletal diseases (MSDS) among medical practitioners. In the field of barrier free design in architecture and space, YOLOv11 is used to build a spatial risk diagnosis framework by combining attention mechanism [25]. Through in-depth analysis of human posture angle (PA) and complex behavioral interactions such as falls and wheelchairs, it provides a quantitative evaluation basis for data-driven indoor space safety optimization [26]. In addition, in the Industrial Ergonomics scene, YOLOv11 has also been innovatively applied to human body area detection in thermal imaging environment to assist enterprises in conducting non-contact ergonomic stress assessment in complex or harsh working environment [27]; At the same time, the improved lightweight network based on YOLOv11-Pose (such as lsp-yolo) further realizes the daily bad sitting posture recognition with high precision and low delay on edge devices with limited computing resources [28]. These cutting-edge pieces of literature and applications jointly show that YOLOv11 is promoting the profound transformation of ergonomics evaluation from the traditional static and artificial subjective observation to the real-time, objective and automated deep learning visual paradigm [29,30].

In recent years, aiming at the inherent depth ambiguity and self occlusion problems in monocular 3D human pose estimation, the Multi-Hypothesis Transformer (MHFormer) architecture has been proposed, which regards 3D pose reconstruction as an inverse problem with multiple feasible solutions [31]. The core of MHFormer lies in its unique three-stage processing mechanism: firstly, multiple reasonable spatial pose hypotheses are extracted and initialized through the multiple hypothesis generation (MHG) module, and then through the self hypothesis refinement (SHR) and cross hypothesis interaction (CHI), each hypothesis is processed independently and the feature relationship is established, and finally the high-precision 3D pose of a single human body is combined and output [16,32]. With its excellent performance on benchmark datasets such as Human3.6M and MPI-INF-3DHP, MHFormer has established its position as the mainstream benchmark for 3D attitude estimation, but it also triggered a discussion on the calculation cost of long sequence input, prompting subsequent research to further explore the lightweight alternative of single frame combined with context [33]. With the deepening of research, MHFormer’s “multi hypothesis” concept has inspired the expansion and application of a large number of cutting-edge pieces of literature. For example, researchers have combined the diffusion model with the multi hypothesis aggregation mechanism (JPMA) to further improve the anti noise ability and accuracy of probabilistic 3D pose estimation in complex scenes [34]; other studies have proposed a spatio-temporal frequency domain adaptive hierarchical fusion Transformer (such as FSA-HFFormer) for complex high-frequency actions, which further reduces the estimation error of MHFormer under extreme attitude through multi domain information interaction [35]. In addition, this high-quality 3D motion representation framework has also been innovatively introduced into real application scenarios to jointly solve complex human activity modeling tasks such as 3D human posture analysis and energy consumption assessment (EEE) [36]. MHFormer not only breaks the limitations of traditional deterministic single point mapping with the paradigm of multi hypothesis generation, but also lays a solid theoretical foundation for the subsequent visual capture and objective behavior analysis of complex space based on Transformer [37,38,39].

In the design of barrier free furniture, the low hinged door has defects and risks for wheelchair users. In the field of barrier free furniture design, the traditional low hinged door, also known as revolving door or swing door, is generally regarded as a design with obvious defects. In the process of opening and closing the door, it will bring greater mechanical burden to the body of wheelchair users. Different from the drawer or shelf that is pulled directly, when the revolving door is opened, it will form a fan-shaped space obstacle area with the hinge as the center and sweeping outward, which requires the user to make complex body avoidance actions. In order to avoid the moving range of the door, users often need to move their bodies backward or sideways [40]. After the door is opened, the user also needs to lean into the depths of the cabinet to pick up and place items. This action usually requires a large bending in a limited space and twisting the body at the same time. This compound posture of bending and twisting will significantly increase the shear force on the lumbar intervertebral disc and force the shoulder joint in an unnatural posture. In the long run, it is easy to cause the strain of the rotator cuff tendon [41]. In contrast, the full pull-out drawer can directly deliver the items in the cabinet to the user, so that people do not need to lean in and reach for things. Therefore, the user can maintain a healthier and more ergonomic sitting posture, and the spine is in a natural alignment state [42].

As for the optimal operating height of the cabinet, the concept of “vertical accessibility” was proposed by ergonomics research. Although the Americans with Disabilities Act (ADA) recommends that the height of the cupboard worktop should generally not exceed 86 cm, further research has found that the optimal height for goods storage and daily operation is not exactly the same. For people who operate in a sitting position, the range from chest to shoulder, about 90 to 110 cm, is the core of the true “neutral accessibility zone” [43]. Within this height range, wheelchair users can naturally bend their elbows (about 90 to 100 degrees) to take things, which can effectively avoid two kinds of body injury positions: one is the collapse of the torso caused by bending down to reach low objects, and the other is the excessive abduction of the shoulders caused by raising their hands to reach high objects (that is, the arm is raised more than 90 degrees). This excessive abduction of the arm is a high-risk factor that causes the supraspinatus tendon to be impacted and then cause injury [44]. Therefore, the design guide increasingly emphasizes that the common storage space is concentrated in this “golden area”, which is more important than simply considering the absolute height of the worktable. In addition, the force required to open the cabinet door must be controlled within 22 newtons, and the style of the door handle must also conform to the principle of closed fist operation (such as using a U-shaped handle), so as to take care of users with weak fine activity ability of the hand [45].

A single intervention is often not enough to completely eliminate musculoskeletal risk. The systematic research and analysis pointed out that in order to reduce the risk most effectively, comprehensive strategies must be adopted, that is, the combination of “engineering control” (such as modifying the physical environment) and “management control” (such as training users). Research shows that relying only on environmental modification will directly reduce the physical load. For example, the installation of automatic doors or height adjustable cabinets helps to improve. However, due to long-term habitual movements, users often continue to use the wrong posture mode [46]. In contrast, if we can combine biomechanical principles and provide targeted training to users, we can teach them how to adjust the position of wheelchairs and use upper limb strength more rationally. The actual data show that this comprehensive intervention method combining environmental transformation and skill education can effectively reduce the degree of musculoskeletal pain of users, that is, the score measured by visual analogue scale (VAS). The long-term effect of this combined method is much better than the improvement of a single dimension [47].

The current ergonomic risk assessment methods for wheelchair users mainly rely on traditional observation technologies such as RULA and Reba. These methods require manual analysis by trained ergonomic experts [48,49]. Although the effectiveness of these mature methods has been verified in different populations, there are still some limitations when applied to wheelchair users: (1) this assessment is very dependent on manual scoring, which is time-consuming and labor-consuming, and it is difficult to be popularized and applied in a wide range or daily environment; (2) It is easy to ignore some subtle postural changes that are important in medicine, because the scoring method is graded; (3) The evaluation process largely depends on the subjective judgment of experts, and different experts are prone to score differences; (4) This method cannot provide real-time data feedback, so it is difficult to give effective intervention guidance at the first time.

Recent breakthrough advances in computer vision and deep learning technologies have unveiled unprecedented opportunities for automated pose estimation and ergonomic risk assessment. Vision-based approaches offer compelling advantages including non-intrusiveness, cost-effectiveness, and enhanced scalability compared to traditional sensor-based systems. However, existing pose estimation methodologies encounter substantial challenges when applied to wheelchair user populations, including: (1) complex occlusion effects caused by wheelchair structural components and self-occlusion during reaching tasks, (2) depth ambiguity inherent in monocular vision systems, (3) limited availability of training datasets representing wheelchair user populations, and (4) difficulty in accurately capturing fine-grained joint movements critical for comprehensive ergonomic assessment.

Existing studies on ergonomic assessment of wheelchair users have primarily focused on general upper-limb activities or propulsion tasks, with relatively limited attention given to interaction with household furniture systems such as cabinets and storage units. In the context of accessibility design, prior research has examined reachability, storage height, and spatial layout based on anthropometric measurements and static posture evaluation [2]. However, these approaches typically rely on manual observation or simplified task assumptions, making it difficult to capture the dynamic changes in biomechanical load during real task execution [8]. In particular, cabinet interaction often involves complex movements such as reaching, trunk flexion, and joint compensation under constrained spaces, which are not adequately represented in traditional assessment frameworks. Moreover, existing ergonomic evaluation methods rarely incorporate automated vision-based pose estimation or continuous risk modeling, limiting their applicability in real-world assistive environments. Therefore, there remains a need for an automated and dynamic ergonomic assessment framework that can accurately capture posture-related risks during cabinet interaction tasks for wheelchair users.

To systematically address these multifaceted challenges, this research presents a comprehensive computational framework that delivers the following key contributions:

Technical Innovation: We propose a novel cascaded architecture that synergistically combines YOLOv11 object detection with Multi-Hypothesis Transformer (MHFormer) technology for robust 3D pose estimation in wheelchair operation scenarios. The YOLOv11 component leverages the proven YOLO architectural family [50], while the pose lifting mechanism utilizes the advanced MHFormer framework [16].

Methodological Advancement: We introduce an innovative fuzzy logic-enhanced RULA assessment system that systematically addresses the discrete scoring limitations inherent in traditional methodologies [51]. Through the implementation of sophisticated trapezoidal membership functions and centroid-based defuzzification algorithms, our approach enables continuous risk quantification that more accurately captures the gradual nature of postural risk variations during dynamic operational sequences.

Comprehensive Validation and Practical Application: Through rigorous experimental validation involving 30 participants across five distinct cabinet operation scenarios, we demonstrate substantial improvements in joint angle estimation accuracy (RMSE: 7.5° versus 18.6° for Kinect v2) while providing a practical, deployable framework for real-time risk assessment in residential environments.

The organizational structure of this paper proceeds as follows: Section 2 provides a comprehensive review of related work in pose estimation and ergonomic assessment methodologies. Section 3 presents our proposed methodology, including detailed technical specifications of each system component. Section 4 describes the experimental design, implementation details, and validation results. Section 5 discusses the broader implications, clinical relevance, and acknowledged limitations of our approach, while Section 6 concludes with future research directions and potential extensions.

## 2. Materials and Methods

### 2.1. System Architecture Overview

**Figure 1:**
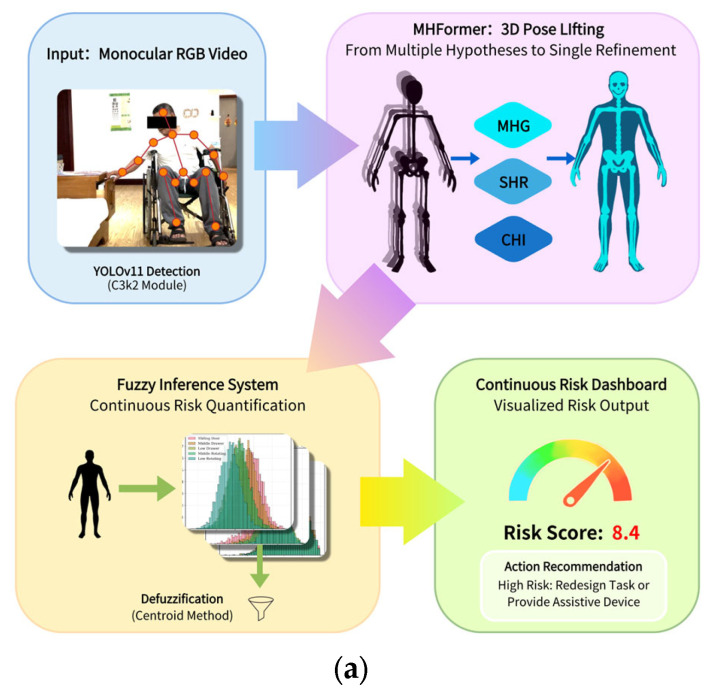

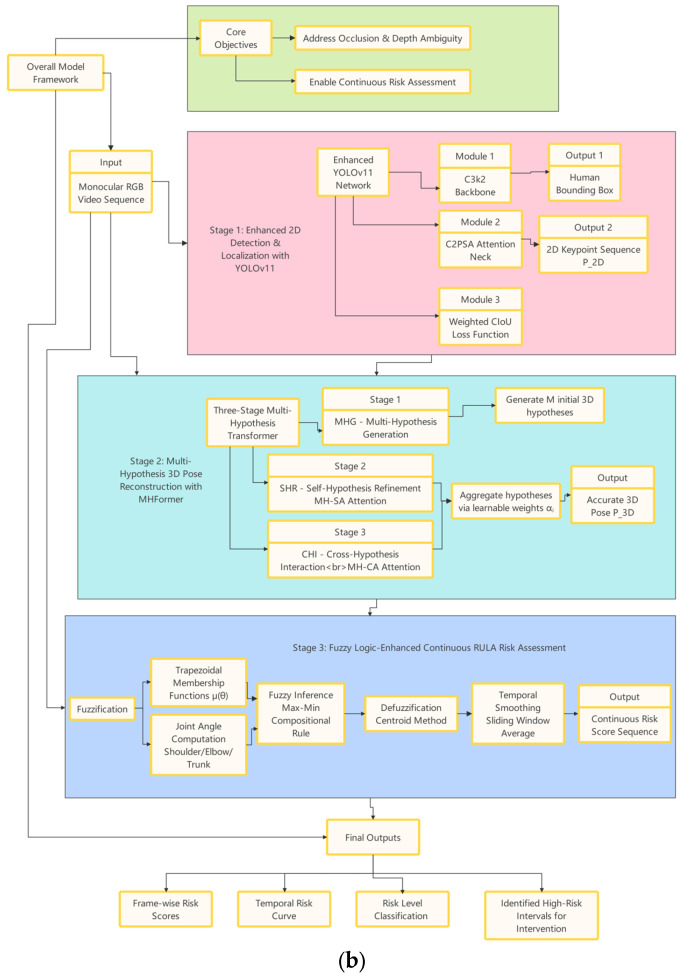
Overall architecture and workflow of the proposed automated ergonomic assessment system. (**a**) Hierarchical system architecture showing the three major computational stages: human detection using YOLOv11, 2D-to-3D pose reconstruction using MHFormer, and ergonomic risk evaluation using RULA with fuzzy logic enhancement. (**b**) Workflow of the proposed system, illustrating the complete data flow from monocular RGB video input to continuous risk score output, with emphasis on the multi-hypothesis 3D pose generation strategy and the trapezoidal membership-based continuous risk quantification method.

To address the challenges of posture reconstruction and ergonomic risk evaluation in wheelchair users during cabinet operation tasks, an automated vision-based assessment framework was developed in this study. This section describes the overall system architecture, data flow, and the main computational modules of the proposed method. The overall architecture and workflow of the framework are illustrated in Figure 1.

The method proposed in this study includes a multi-step calculation process, and its original design purpose is to solve the special difficulties faced by wheelchair users in three-dimensional pose estimation and ergonomic risk assessment. The overall structure of the system consists of three core parts: the first part uses YOLOv11 model to detect human body and extract two-dimensional pose information; In the second part, the two-dimensional pose is reconstructed into three-dimensional pose by MFformer model; The third part uses the RULA method with fuzzy logic to evaluate the ergonomic risk of posture [52,53,54,55,56].

The input data of this system are the monocular RGB video sequence taken by wheelchair users when they perform cabinet operation tasks. The YOLOv11 detector first accurately locates the human body target and extracts two-dimensional key points, and then is processed by the mhformer network to reconstruct the accurate three-dimensional joint coordinates. Finally, the 3D attitude data are input into the enhanced RULA evaluation module to generate continuous risk scores and evidence-based safety recommendations.

### 2.2. YOLOv11-Based Human Detection and 2D Pose Estimation

#### 2.2.1. Architecture Design

In the ergonomic analysis of wheelchair users, the detection model used must be able to accurately identify and extract the characteristics of the relevant parts of the human body from the complex environmental background composed of wheelchairs, cabinets, etc. To meet this demand, this paper selects the model YOLOv11 [50] and specifically adjusts and optimizes the model for the specific scenario of users’ operation of cabinets. The YOLOv11 model was initialized using pre-trained weights from the COCO dataset and subsequently fine-tuned on the self-constructed dataset collected in this study, enabling improved detection performance under wheelchair-specific conditions, particularly in scenarios involving seated posture variations and occlusion caused by wheelchair structures.

The network analyzes the RGB video images of wheelchair users through three core modules, which are the backbone network responsible for feature extraction, the neck network responsible for feature fusion and the head network responsible for final prediction.

Backbone network is the core of feature extraction of the whole model. Its main task is to accurately extract the features of users’ upper limbs in the complex background including wheelchair handles, wheels and other distractions. This paper introduces an innovative C3k2 module, which replaces the original C2f module in yolov8, so as to enhance the ability of the model to express features in complex environments. The C3k2 module is designed with a cross phase partial (CSP) bottleneck structure including double small convolution kernels. This innovative design enables the network to maintain sufficient gradient information flow in the face of the complex visual overlap between the user’s body and wheelchair. Its mathematical expression is:

(1) $$\begin{array}{c} Y=C3k2(X)={Conv}_{3\times 3}({Conv}_{3\times 3}(X))+X \end{array}$$

where $X\in R^{B\times H\times W\times C}$ represents the input feature tensor extracted from the operation frames (batch size B, spatial dimensions H×W, and C channels). In our application, X encodes both the user’s posture and the surrounding cabinet/wheelchair structures, and the residual connection in Equation (1) ensures that fine-grained details of joint positions are preserved during deep feature extraction.

When operating the cabinet, the main difficulty is that during the process of reaching out, the user’s arm is often blocked by the cabinet door or the armrest of the wheelchair. To alleviate this problem, this paper introduces an advanced C2PSA (cross phase partial spatial attention) module in the neck of the network. Different from the traditional convolution operation, C2PSA module can guide the model to focus on those visible human parts by introducing the spatial attention mechanism, and weaken the interference information from the furniture background such as cabinets. The attention weight matrix A is computed as:

(2) $$\begin{array}{c} A=Softmax\left(\frac{QK^{T}}{\sqrt{d_{k}}}\right)V \end{array}$$

where Q, K, and V represent the feature matrices derived from the backbone’s output, and $d_{k}$ denotes the key dimension. In the system we actually built, this mechanism enables the model to dynamically allocate calculation weights according to the correlation between different regions in the image and the target, that is, give a higher attention weight (A) to the region where the human joints are located, and give a lower weight to the stationary wheelchair and cabinet background. When the arm is partially occluded due to the user’s actions such as “holding up” or “rotating the door”, the network can still accurately predict the position of the occluded limb with high confidence.

#### 2.2.2. Loss Function Enhancement

The traditional boundary box regression loss function is usually difficult to accurately describe the special geometric shape of wheelchair users, because in this case, a boundary box needs to frame the deformable human body and the shape fixed wheelchair structure at the same time. When the user is in a dynamic operation scene, such as leaning forward to reach a lower drawer, the length width ratio of the target bounding box will change significantly from that in the normal sitting position. In order to solve this problem, this study uses the weighted complete intersection union ratio (CIoU) loss function [51]. Different from the standard intersection union ratio calculation method, CIoU not only pays attention to the overlapping degree of the frame, but also minimizes the normalized distance between the center points of the two bounding boxes, and forces the length width ratio of the two to be consistent, so that even when the user’s body is partially blocked by the cabinet door, it can still obtain stable and accurate positioning effect.

The CIoU loss is mathematically formulated as:

(3) $$\begin{array}{c} L_{CIoU}=1-IoU+\frac{\rho^{2}(b,b^{gt})}{c^{2}}+\alpha v \end{array}$$

In the formula, $\rho (b,\;b^{gt})$ represents the straight-line distance between the predicted center point of the bounding box and the center point of the real labeled bounding box, while c refers to the diagonal length of the smallest circumscribed rectangle that can contain both bounding boxes. The key parameter v is used to measure the similarity between the predicted frame and the real frame in the length width ratio. This enables the model to better adapt to the obvious changes in the shape of the bounding box caused by posture changes when performing tasks such as two handed operation or lateral retrieval:

(4) $$\begin{array}{c} v=\frac{4}{\pi^{2}}{\left(\arctan\frac{w^{gt}}{h^{gt}}-\arctan\frac{w}{h}\right)}^{2} \end{array}$$

To balance the dual objectives of robust human localization and precise joint identification, the total loss function $L_{total}$ linearly combines the bounding box regression loss $\left(L_{CIoU}\right)$ and the keypoint detection loss $\left(L_{keypoint}\right)$:

(5) $$\begin{array}{c} L_{total}=\lambda_{1}L_{CIoU}+\lambda_{2}L_{keypoint} \end{array}$$

The super parameters $\lambda_{1}$ and $\lambda_{2}$ in this study are adjusted and optimized based on experimental experience. The purpose is to ensure that the model can give priority to the detection effect of the composite entity of “users and wheelchairs” in complex application scenarios. As shown in Table 2, the system gives a higher weight to the CIoU index to ensure that the detection window can accurately frame the user before the start of attitude estimation.

In addition to the loss-related hyperparameters described above, the overall training configuration of the proposed framework also plays an important role in model performance and stability. To improve the transparency and reproducibility of the proposed method, the main training hyperparameters, including input resolution, batch size, learning rate, optimizer, and training epochs, are summarized in Table 3.

**Figure 2:**
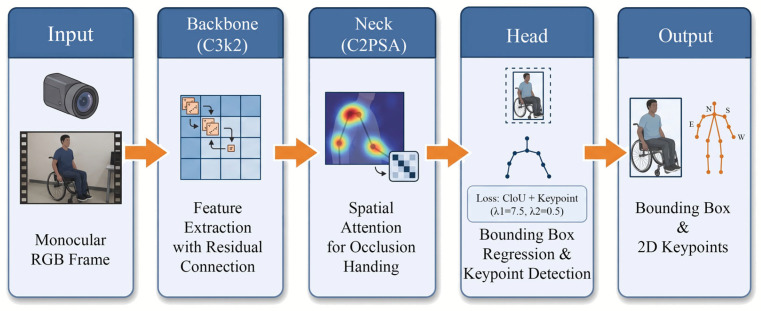
Workflow of YOLOv11-based human detection and 2D pose estimation module.

The complete workflow of the YOLOv11-based 2D pose estimation module is illustrated in Figure 2.

### 2.3. MHFormer-Based 3D Pose Reconstruction

#### 2.3.1. Multi-Hypothesis Framework

**Figure 3:**
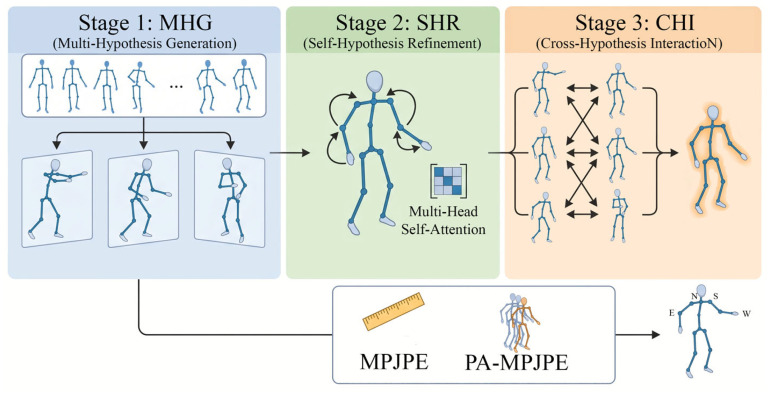
Workflow of MHFormer-based 3D pose reconstruction from 2D joint sequences.

After the YOLOv11 detector obtains the two-dimensional coordinates of human joints from the image, the system needs to deal with a fundamental challenge inherent in monocular vision technology, that is, the fuzziness of depth information. Taking the action of wheelchair users bending over to open the lower drawer as an example, the changes in arm length observed in the two-dimensional image may correspond to a variety of different three-dimensional space postures in Physics: for example, the elbow bending may lead to visual shortening, or the whole arm may be straight towards the camera. In order to solve this ambiguity problem, MHFormer model is adopted in this study. The model transforms the process of three-dimensional attitude estimation from a regression problem that directly outputs a single determined result to a “multi hypothesis” optimization problem that seeks the optimal solution from a variety of possibilities [16]. The framework accepts the sequence of 2D keypoints generated by YOLOv11 as input, formally defined as $P_{2D}\in R^{T\times J\times 2}$, where T represents the temporal window size corresponding to operation phases (approach, engagement, and withdrawal), J denotes the number of anatomical joints (including critical ergonomic landmarks like shoulders and elbows), and the last dimension corresponds to the (x,y) coordinates in the image plane. The principle of this mechanism is to generate a number of three-dimensional postures that are physically possible as alternative assumptions, and then screen them for optimization. This method is particularly effective when dealing with actions that are prone to self occlusion, such as “revolving door opening”. When the user’s arm frequently crosses the torso, the system can effectively distinguish between complex compensatory actions and standard stretching actions. Figure 3 provides an overview of the MHFormer-based 3D pose reconstruction pipeline, detailing the three-stage multi-hypothesis refinement process.

#### 2.3.2. Stage 1: Multi-Hypothesis Generation (MHG)

In order to fundamentally solve the problem of ambiguous depth information mentioned above, the MHG module is introduced in this study, which acts as a starter to provide initial input for subsequent calculations. When wheelchair users extend their arms to operate the sliding door, the exact depth of the wrist relative to the shoulder in the two-dimensional plane is uncertain. The MHG module’s response strategy is to generate a variety of different initial three-dimensional poses as candidates to cover various possibilities, so as to ensure that the real pose must be included in the search range formed by these candidate schemes:

(6) $$\begin{array}{c} H^{\left(0\right)}=\{h_{1}^{\left(0\right)},h_{2}^{\left(0\right)},\ldots ,h_{M}^{\left(0\right)}\} \end{array}$$

where each hypothesis $h_{i}^{\left(0\right)}\in R^{T\times J\times d}$ represents a plausible biomechanical configuration of the user’s upper body. These hypotheses are generated by projecting the YOLO-detected 2D keypoints ($P_{2D}$) into a high-dimensional feature space via learnable transformations:

(7) $$\begin{array}{c} h_{i}^{\left(0\right)}=LayerNorm(W_{i}P_{2D}+b_{i}) \end{array}$$

Here, the trainable parameter $W_{i}\in R^{d\times 2}$ and the offset term $b_{i}\in R^{d}$ enable the network to map different two-dimensional joint patterns to a variety of spatial possibilities. In the face of the same two-dimensional image input, a three-dimensional hypothesis generated by the network may be manifested as the obvious bending action made by the user to adapt to the wheelchair activity space; Another equally reasonable assumption may be the different limb shapes driven by the outward expansion of the shoulders. Generating this diverse postural hypothesis is of great significance for capturing the complex compensatory movement patterns unique to wheelchair users.

#### 2.3.3. Stage 2: Self-Hypothesis Refinement (SHR)

After generating diversified initial assumptions, the system will enter the self hypothesis optimization (SHR) stage, in which the three-dimensional posture of wheelchair users is fine adjusted through repeated iterative calculations to make it more reasonable and accurate. This optimization process is at frames 30 to 120 of the whole action, which is the key stage for the user to actually interact with the cabinet and perform the operation. At this stage, users often make complex gestures such as excessive shoulder abduction or large torso twisting in order to complete actions such as crossing the obstacles inside the cabinet. In order to systematically optimize these complex candidate postures without losing the diversity necessary to reflect the unique adaptation of different individuals, this study introduced the multi hypothesis self attention mechanism (MH-SA). For each hypothesis $h_{i}$, the self attention calculation formula is expressed as:

(8) $$\begin{array}{c} MH-SA(h_{i})=Attention(Q_{i},K_{i},V_{i}) \end{array}$$

Among them, the calculation formulas of query matrix, key matrix and value matrix representing the internal biomechanical dependence between anatomical joints are:

(9) $$\begin{array}{ll} Q_{i} & =h_{i}W_{Q} \end{array}$$

(10) $$\begin{array}{ll} K_{i} & =h_{i}W_{K} \end{array}$$

(11) $$\begin{array}{ll} V_{i} & =h_{i}W_{V} \end{array}$$

Through the above matrix calculation, the module can digitally encode the spatial position relationship between key joint chains such as neck, shoulder and elbow. This step is very important for the subsequent RULA evaluation to accurately calculate the angles of each joint. Finally, in order to strengthen the information sharing among different postures and avoid the model finally getting only one kind of depth information judgment that may not be accurate, all optimized postures will be sent to the hypothesis hybrid multilayer perceptron (*MLP*) module for further integration and processing:

(12) $$\begin{array}{c} h_{i}^{\left(1\right)}=MLP{\left(Concat(MH-SA(h_{1}),\ldots ,MH-SA(h_{M}))\right)}_{i} \end{array}$$

This network structure design ensures that the model can learn from the common law reached by multiple three-dimensional postures, so as to form a more stable and reliable digital description of the motion trajectory of the upper limb in the process of dynamic interaction between the user and the cabinet.

#### 2.3.4. Stage 3: Cross-Hypothesis Interaction (CHI)

In the final stage of 3D reconstruction, the system will enable the cross hypothesis interaction (CHI) module, which is used as a global optimizer to ensure that the final generated 3D posture conforms to the rationality and limitations of wheelchair users in biomechanics. This optimization step is at frames 30 to 120 of the whole action, that is, the stage when the user actually touches the cabinet and executes the operation. At this stage, the user’s limbs tend to extend to the maximum extent, and the mutual shielding between body parts is also the most serious. To systematically solve the residual deep inconsistency, CHI module promotes the comprehensive information exchange between different hypotheses through the multi hypothesis cross attention (MH-CA) mechanism:

(13) $$\begin{array}{c} MH-CA(h_{i},h_{j},h_{k})=Attention(Q_{i},K_{j},V_{k}) \end{array}$$

The mechanism can identify and eliminate the depth estimation results that are physically impossible for wheelchair users by comparing and associating the spatial information of multiple different candidate postures. The final three-dimensional pose estimation, which provides a coordinate basis for the calculation of joint angles such as shoulder flexion and elbow flexion, is obtained through learnable weighted aggregation:

(14) $$\begin{array}{c} P_{3D}=\sum_{i=1}^{M}\alpha_{i}h_{i}^{\left(final\right)} \end{array}$$

The attention weight $\alpha_{i}$ is calculated by normalizing the confidence scores of each hypothesis automatically learned by the network through a softmax function. This mechanism ensures that the system can fuse multiple attitude assumptions in an optimal way. This stable and reliable hypothesis integration method enables the framework to accurately identify high-risk ergonomic operation conditions (RULA>3.0), such as the situation that users continue to bear ergonomic pressure during low revolving door operation. By fusing the spatial attitude information with the highest confidence, the cross hypothesis interaction (CHI) module makes the estimation accuracy of the joint angle of the whole system reach the level that the average root mean square error (RMSE) is only 7.5 degrees. Compared with the traditional depth sensor benchmark method, this result achieves a significant improvement of up to 60% in the accuracy of joint angle estimation.

#### 2.3.5. Training Objective

In order to make the MHFormer model accurately reconstruct the three-dimensional motion trajectory of wheelchair users when operating the cupboard, we used two error evaluation indexes when training the model: one is the mean joint position error (MPJPE), and the other is the joint position error after procrustes alignment (PA-MPJPE). The advantage of this training strategy is that, on the one hand, it can meet the needs of accurate positioning of the absolute position of the joint in space, and on the other hand, it can ensure that the relative position relationship between the relevant nodes is consistent during the fine joint angle analysis. The loss function of the strategy is specifically defined as follows:

(15) $$\begin{array}{c} L_{MHFormer}=L_{MPJPE}+\lambda L_{PA-MPJPE} \end{array}$$

The first term, $L_{MPJPE}$, serves to minimize the absolute displacement of J joints across T frames, ensuring the reconstructed pose remains spatially grounded within the functional interaction zone of the furniture:

(16) $$L_{MPJPE}\&=\frac{1}{TJ}\sum_{t=1}^{T}\sum_{j=1}^{j=1}\|\;p_{t,j}^{pred}-p_{t,j}^{gt}{\|}_{2}$$

The second term, $L_{PA-MPJPE}$, further refines the relative anatomical structure through Procrustes alignment, which is essential for obtaining the precise 3D skeletal data used to compute shoulder and elbow flexion angles for the RULA assessment:

(17) $$L_{PA-MPJPE}\&=\frac{1}{TJ}\sum_{t=1}^{T}\sum_{j=1}^{J}\|Procrustes(P_{t}^{pred})-P_{t}^{gt}{\|}_{2}$$

By optimizing these two objectives, the model achieves the high accuracy required to reduce the average RMSE to 7.5°, which provides a reliable basis for identifying high-risk ergonomic stressors in daily furniture use. The reference ground truth for joint angle estimation was obtained through manual annotation and geometric computation. Specifically, key anatomical landmarks, including the shoulder, elbow, and wrist joints, were manually labeled for selected frames using an annotation tool. Based on these labeled keypoints, joint angles were calculated using vector-based methods according to standard kinematic definitions. This procedure ensures that the ground truth values are consistent with the biomechanical definitions used in ergonomic assessment.

### 2.4. Fuzzy Logic-Enhanced RULA Assessment

#### 2.4.1. Traditional RULA Limitations

In order to establish a set of measurement standards for ergonomic evaluation, this study uses rapid upper limb assessment (RULA) as the core evaluation system. This assessment tool was developed by McAtamney and Corlett. Its basic idea is to divide the posture of the human body into two different groups, so as to assess the risk of musculoskeletal injury to individuals at work.

Group A: Evaluates the upper limbs, including the upper arms, lower arms, and wrists.

Group B: Focuses on the neck, trunk, and legs.

In this research on cabinet operation, the 3D joint point coordinates reconstructed by the method described in Section 2.3 above will be given a discrete score according to the traditional evaluation process of RULA; Then, these scores will be comprehensively calculated through a series of comparison tables, and finally a total score between 1 and 7 will be obtained. This final score corresponds to four levels of action requirements: if the score is 1 or 2, it means that the current posture is in an acceptable range; If the score reaches 7, it means that engineering improvement or change of operation behavior must be taken immediately to reduce ergonomic risk.

The traditional RULA evaluation method adopts a discrete scoring method, which artificially divides the original continuously changing joint angle value into several fixed intervals, and then carries out the grade evaluation [48]. Fundamentally speaking, this treatment method cannot truly reflect the gradual and continuity of ergonomic risk in the process of joint angle change; It artificially creates a fault in the risk assessment, which may cause the assessment results to be inconsistent with the actual stress situation of the human body in biomechanics.

#### 2.4.2. Fuzzy Membership Function Design

**Figure 4:**
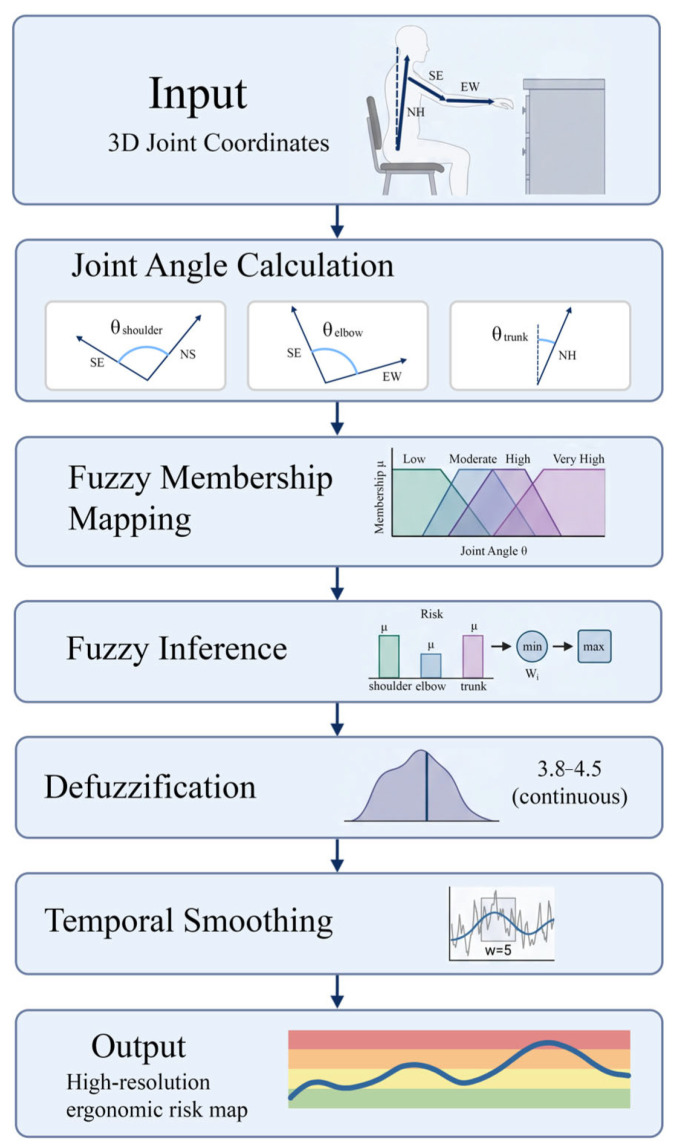
Workflow of fuzzy logic-enhanced RULA assessment for continuous ergonomic risk scoring.

In order to fundamentally solve the problem of discontinuous scoring criteria in the traditional RULA method, a fuzzy logic method based on trapezoidal membership function is introduced to realize the continuous quantitative evaluation of ergonomic risk. The overall workflow of the proposed fuzzy logic-enhanced RULA assessment is depicted in Figure 4, highlighting the transformation from joint angles to a continuous, smoothed risk score.

Research and observation show that all kinds of movements of wheelchair users, such as the process of gradually leaning forward from neutral sitting position to the maximum when operating the middle drawer, are smooth and continuous in nature, rather than jumping and discontinuous. By introducing fuzzy logic, the system can effectively describe how the work load of the human body gradually increases when the joint angle θ changes continuously and gradually exceeds each threshold in the traditional RULA score.

For any specific joint angle θ (e.g., shoulder or elbow flexion), its membership degree in risk category $R_{i}$ is mathematically defined as:

(18) $$\begin{array}{c} \mu_{R_{i}}(\theta )=\left\{\begin{array}{ll} 0 & if\;\theta <a_{i}\;or\;\theta >d_{i}\\ \frac{\theta -a_{i}}{b_{i}-a_{i}} & if\;a_{i}\;\leq \;\theta <b_{i}\\ 1 & if\;b_{i}\;\leq \;\theta \leq c_{i}\\ \frac{d_{i}-\theta }{d_{i}-c_{i}} & if\;c_{i}<\theta \leq d_{i} \end{array}\right. \end{array}$$

By setting the characteristic parameters $\left(a_{i},b_{i},c_{i},d_{i}\right)$, we construct overlapping trapezoidal regions for each risk level as the basis of fuzzy evaluation. This design enables the entire assessment framework to sensitively capture the subtle risk fluctuations that occur when the body load reaches its peak at the critical stage (frames 30–120) when the user actually touches and operates the cabinet. For example, when operating cabinets in lower positions, the average RULA score tends to exceed 4.0. At this time, the membership function we designed can ensure that the risk level is a smooth transition from “high” (corresponding to the original score of 3) to “extremely high” (corresponding to the original score of 4) in mathematical expression. Compared with the traditional method of simply setting a fixed threshold, this method can more accurately reflect the real biomechanical process of gradual accumulation of postural pressure in continuous motion.

#### 2.4.3. Joint-Specific Risk Computation

Based on the accurate three-dimensional coordinate data ($P_{3D}$) reconstructed by the MHFormer module, this study further calculated the biomechanical direction vector of the main joints of the human body. This calculation step acts as a bridge, effectively transforming the original computer vision output results into interpretable indexes with ergonomic analysis value.

In the operation involving rotational mechanics, it is necessary to calculate the flexion and extension angle $\theta_{shoulder}$. We use the direction vector derived from the neck (N), shoulder (S), and elbow (E) keys:

(19) $$\begin{array}{c} \theta_{shoulder}=\arccos\left(\frac{\vec{NS}\cdot \vec{SE}}{\left|\vec{NS}|\cdot |\vec{SE}\right|}\right) \end{array}$$

This calculation is pivotal for identifying the increased shoulder depression observed in low-positioned operations (peaks around 130–140°).

Our results showed that the elbow angle exhibited the highest correlation with ergonomic risk score among the analyzed joint variables (r = 0.311). However, this value indicates a weak to moderate positive relationship rather than a strong predictive effect. This suggests that elbow posture contributes to ergonomic risk, but should be interpreted as one of several contributing factors rather than a dominant predictor.

To further interpret this relationship, we analyzed the biomechanical demands associated with lower-drawer operations. During these tasks, users are required to maintain a stable grip while extending the arm downward, which typically results in increased elbow flexion, with observed angles ranging from approximately 110° to 130°. Such postural configurations are associated with increased biomechanical load on the upper limb, thereby contributing to elevated ergonomic risk scores.

(20) $$\theta_{elbow}\&=\arccos\left(\frac{\vec{SE}\cdot \vec{EW}}{\left|\vec{SE}|\cdot |\vec{EW}\right|}\right)$$

where $\vec{SE}$ and $\vec{EW}$ represent the shoulder-to-elbow and elbow-to-wrist vectors, respectively.

Different from standing operators, users in wheelchairs mainly rely on the action of bending the torso in order to increase the space range that their arms can reach, especially when the handle of the cabinet is installed at a relatively low position. We quantify this compensation strategy by calculating the deviation between the torso vector $\vec{NH}$ (Neck-to-Hip) and the virtual vertical reference $\vec{VR}$:

(21) $$\theta_{trunk}\&=\arccos\left(\frac{\vec{NH}\cdot \vec{VR}}{\left|\vec{NH}|\cdot |\vec{VR}\right|}\right)$$

This indicator can effectively monitor the relative position relationship between the user’s torso and thigh. The data show that the relationship remains stable (165–175), unless extreme stretching is required.

#### 2.4.4. Fuzzy Inference and Defuzzification

In order to extract an ergonomic feature reflecting the overall situation from the evaluation results of a single joint, the fuzzy reasoning system in this study will comprehensively integrate the risks of each joint constituting the upper limb motion chain. As the core of decision-making, the function of this module is to convert the measured biomechanical data into a risk level with clinical significance. For wheelchair users who are engaged in multiple tasks such as sliding door operation, different joints may present different risk levels at the same time. To this end, we use the max-min combination rule to aggregate individual joint risks:

(22) $$\begin{array}{c} \mu_{total}(r)=\underset{i}{\max}\min(\mu_{R_{i}}(\theta_{i}),w_{i}) \end{array}$$

where $w_{i}$ represents the clinical importance weight for joint i (e.g., shoulder, elbow, and trunk). In our framework, the role of the min operator is to ensure that the final risk assessment results are restricted by the credibility of each joint’s respective assessment results, while the max operator reflects the “bottleneck” principle in Ergonomics—the overall posture risk is often determined by the anatomical structure with the greatest force. This is very typical when operating the cabinet handle at a lower position: even though the user’s torso is still supported by the wheelchair back, the risk is small, but just because the elbow is excessively bent, it is enough to push up the overall risk score.

In order to convert the generated fuzzy aggregate $\mu_{total}(r)$ into an operable and accurate RULA score, we use the centroid defuzzification method:

(23) $$\begin{array}{c} Risk\;Score=\frac{\int r\cdot \mu_{total}(r)\;dr}{\int \mu_{total}(r)\;dr} \end{array}$$

This continuous output enables the system to identify subtle risk changes that cannot be captured by the traditional discrete RULA (score 1–7). In our experimental evaluation of revolving door operation, the defuzzification process realized the identification of high-precision critical moments when the risk score fluctuated between 3.8 and 4.5, thus generating a high-resolution time distribution map of ergonomic pressure in the whole interaction sequence.

#### 2.4.5. Temporal Smoothing

Although the mhformer model can provide advanced human posture reconstruction effect, if we directly use the original risk score ($S_{raw}(t)$) calculated independently for each frame, this value may still produce some transient and unstable fluctuations due to the slight tremor of joints or the rapid movement of limbs during dynamic operation. In our cabinet operation scenes (such as sliding doors or work at height tasks), the temporary occlusion that may exist in the visual environment will cause the 3D joints to flicker slightly between frames. In order to ensure that the final ergonomic evaluation result reflects the continuous physical load of the user, rather than the measurement noise caused by the above reasons, we further use the sliding window smoothing filter to process the original score of the fuzzy reasoning system output, and the calculation method is as follow:

(24) $$\begin{array}{c} S_{smooth}(t)=\frac{1}{w}\sum_{i=0}^{w-1}S_{raw}(t-i) \end{array}$$

where w=5 represents the empirically determined window size. This value is determined based on the experimental data. It has been specially optimized and adjusted for the common body movement speed of wheelchair users in daily operation. By averaging multiple scoring values in a time window, the system can effectively filter out those high-frequency data fluctuations caused by accidental factors, and at the same time completely retain the risk change trend with analytical value in the key stage of actual contact and operation of the cabinet (frames 30–120). Ensuring this stability of the data in the time dimension is crucial for accurately quantifying the sustained body load in tasks such as operating a lower revolving door. Because in the whole process of such tasks, the ergonomic risk is always maintained at a high level. The final score of $S_{smooth}(t)$ obtained by smoothing is used as a core and stable measurement index when comparing different cabinet heights and different types of handles in this study. This provides a reliable data basis for our research conclusions on the cumulative musculoskeletal load of wheelchair users.

### 2.5. Algorithm Summary

Algorithm 1 summarizes our complete pose estimation and risk assessment pipeline.

This method integrates advanced computer vision perception and expert driven ergonomic logic into a coherent end-to-end process. As shown in Algorithm 1, the input of this system is an original video marked V, which records the whole process of interaction between wheelchair users and cabinet furniture. In the first stage, YOLOv11 detector D is responsible for locating the user in each frame and extracting the two-dimensional coordinate point $P_{t}^{2D}$ of his body joints. At the same time, the detector also uses the bounding box (bbox) to cut out the key area (ROI) containing users, so as to minimize the data interference caused by the surrounding furniture background. Then enter the second stage, and the system will input the two-dimensional joint point data $P_{2D}$ of the entire video sequence into mhformer M. The model is responsible for reconstructing the attitude data $P_{3D}$ which is consistent in time and space and has three-dimensional coordinates. This step is very important for accurately capturing the highly sensitive motion trajectory of the shoulder and elbow in the depth direction during the user’s reaching out. In the final stage, this framework will use the RULA system enhanced by fuzzy logic to evaluate the human posture corresponding to each frame. Then, the results of these frame by frame evaluations are smoothed in time, and finally a stable and reliable risk change curve is generated. This curve is used to identify those key pressure points where the ergonomic load reaches the peak in the actual interaction process.
**Algorithm 1.** Wheelchair User Pose Estimation and Risk AssessmentRequire: Video sequence $V=\{I_{1},I_{2},\ldots ,I_{T}\}$ Ensure: Risk scores $\left\{R_{1},R_{2},\ldots ,R_{T}\right\}$ 1: Initialize YOLOv11 detector D,MHFormerM
2: for each frame $I_{t}$ in V do 3: box, $P_{2D}^{t}\leftarrow D(I_{t})$ 4: Crop ROI using bbox 5: end for 6: $P_{2D}=\{P_{2D}^{1},\ldots ,P_{2D}^{T}\}$ 7: $P_{3D}\leftarrow M(P_{2D})$ 8: for each frame t do 9: Compute joint angles: $\left\{\theta_{shoulder}^{t},\theta_{elbow}^{t},\theta_{trunk}^{t}\right\}$ 10: Apply fuzzy membership functions 11:       Perform fuzzy inference 12:       $R_{t}\leftarrow$ Defuzzify using centroid method 13: end for 14: Apply temporal smoothing to $\left\{R_{1},\ldots ,R_{T}\right\}$ 15: return Smoothed risk scores

The unified algorithm can ensure that the system can automatically convert the original visual data into operable health insights, which is highly related to expert evaluation and maintains the computational efficiency required for real-time monitoring of wheelchair accessible environment.

## 3. Results

A total of 30 participants were recruited in this study, including 14 in the experimental group (long-term wheelchair users, wheelchair use time > 3 years) and 16 in the simulation group (healthy participants, after 3 h of wheelchair adaptability training). All participants received anthropometric data collection, and the wheelchair stool surface was used as the support plane. The vertical distance (i.e., sitting height) from the wheelchair stool surface to the top of the head is 89–111 cm (M = 100.02 cm, SD = 5.41 cm). According to Shapiro–Wilk test, the sitting height data conforms to the normal distribution (*p* > 0.05). According to the age classification standard published by the World Health Organization (who), the participants were divided into youth, middle age and old age, as shown in Table 4.

The experimental system consists of four parts: cabinet furniture, wheelchair, camera equipment and experimental environment. Table 5 shows the five types of typical cabinet furniture selected in the experiment.

In the experiment, an iPhone 16 was fixed on a tripod and placed 1.5 m in front of the participant. The front video was taken at 1080 p/30 fps, and the upper limb movement and the opening track of the cabinet door were recorded completely. The right side of the participant’s body is close to the cabinet furniture, and the camera is located on the left side of the experimental device. After pre-experiment, the angle is adjusted to ensure that the key actions are not blocked. The experimental environment simulates the real home scene, the operation area is 3 × 3 M, the ground is flat, and the wheelchair route is fixed. Cover different periods of time and lighting conditions to enhance the robustness of the model.

**Figure 5:**
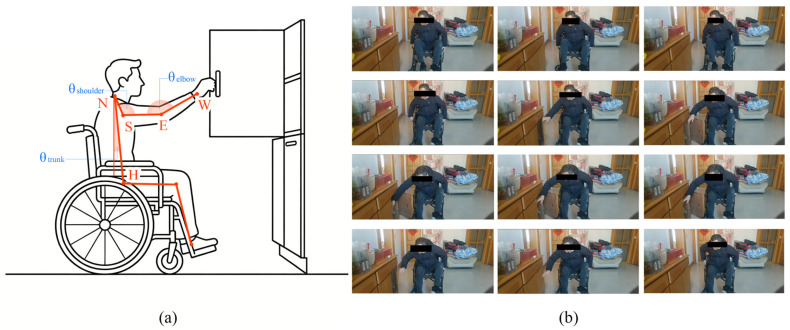
Experimental setup. (**a**) illustrates the definitions of the three joint angles derived from the 3D skeleton: shoulder flexion ($\theta_{shoulder}$), elbow flexion ($\theta_{elbow}$), and trunk inclination ($\theta_{trunk}$). (**b**) shows the real experimental environment and a participant performing the cabinet operation.

To provide an intuitive understanding of the biomechanical parameters used in this study, Figure 5 illustrates the definitions of the three joint angles derived from the three-dimensional skeletal model, along with screenshots from video frames depicting a single operational procedure.

Before the experiment, the participants were prepared for 5 min, and the height of the fixed wheelchair stool was 49.5 cm; each task was repeated three times, and the third piece of the data was taken as the basis for formal record. The implementation process of the experimental task is strictly in accordance with the preset process, and the specific steps are shown in Table 6 below:

A total of approximately 5 h of RGB video were acquired from 30 participants across five cabinet-interaction scenarios. Considering that densely sampled consecutive frames are highly correlated and may increase the risk of model overfitting, the dataset was constructed using a frame-sampling strategy rather than directly using all video frames. Specifically, representative frames were extracted from continuous video sequences at regular intervals, while key frames corresponding to critical action transitions (e.g., hand leaving the wheelchair handrim and returning to it) were additionally retained through manual screening. Following this procedure, 10,000 images were selected from the original video sequences for dataset construction and annotation. To reduce redundancy caused by consecutive frames with minimal motion variation, frames were sampled at fixed intervals. In addition, key frames corresponding to different stages of cabinet interaction tasks (e.g., reaching, manipulation, and return to neutral posture) were deliberately included to ensure sufficient diversity in posture representation. Wheelchair-user joint keypoints were annotated using the CVAT platform.

During dataset construction, diversity across subjects, task types, operation stages, and illumination conditions was considered to improve representativeness and model generalization. The final dataset was divided into training, validation, and test subsets in a 70:15:15 ratio. The training set was used to optimize model parameters, the validation set was used for hyperparameter tuning, and the testing set was reserved for final performance evaluation. To avoid data leakage, samples from the same video sequence were kept within the same subset.

### 3.1. Joint Angle Estimation Accuracy

Compared with the Kinect v2 baseline, the proposed YOLOv11 + MHFormer method achieved lower joint-angle estimation errors across five representative posture patterns involved in cabinet operation tasks. These posture patterns included arm raising, bilateral object holding, arm extension, neck flexion, and forward trunk flexion. Table 7 presents the root mean square error (RMSE) values for the two methods, showing consistent error reduction across all evaluated conditions.

The Kinect v2 baseline was evaluated on the same subset of cabinet-interaction sequences as the proposed method, ensuring a fair comparison under identical task conditions. Because the Kinect v2 data were collected in the same experimental setup, its measurements were also affected by occlusion caused by wheelchair structures, cabinet components, and self-occlusion during reaching tasks. The Kinect-derived joint angles were obtained using the Kinect SDK, and the RMSE was computed using the same joint-angle definitions and evaluation protocol as those used for the proposed method.

The proposed method achieved an average RMSE of 7.5°, compared with 18.6° for Kinect v2, corresponding to an error reduction of approximately 60%. The largest improvements were observed in posture patterns involving greater occlusion and trunk movement, such as forward trunk flexion and bilateral object holding. These results indicate that the proposed vision-based framework provided more accurate joint-angle estimation than Kinect v2 under the evaluated cabinet-operation-related postures.

### 3.2. Temporal Risk Evolution Analysis

**Figure 6:**
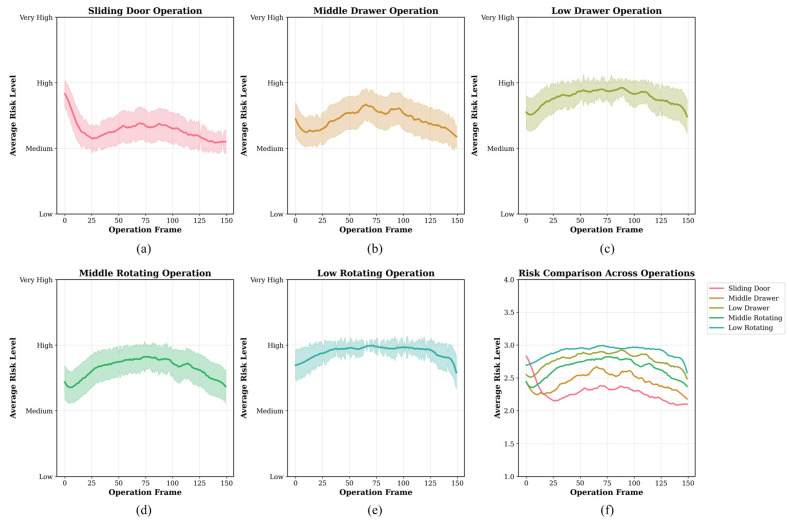
Temporal risk analysis across cabinet operations. Panels (**a**–**e**) present the smoothed mean risk trajectories with confidence intervals for each cabinet-operation task. Panel (**f**) compares the temporal risk profiles across all tasks and shows that low revolving-door and low-drawer operations maintained the highest risk levels during the middle phase of the task. For interpretation, the continuous risk output was categorized into four qualitative levels: low, medium, high, and very high.

Figure 6 illustrates the temporal evolution of ergonomic risk during the five cabinet-operation tasks. Across all task types, the risk trajectories showed a broadly similar overall pattern, indicating a consistent progression of posture-related risk throughout task execution.

All five operations showed a broadly similar three-phase tendency. During the initial phase (approximately frames 0–30), the risk trajectories changed as users moved from the starting posture toward task engagement. During the task-execution phase (frames 30–120), the ergonomic risk remained rather high, suggesting the increased postural stress caused by reaching, trunk flexion, and upper-limb movement. During the return phase (frames 120–150), the risk gradually decreased as the person returned to a more neutral posture.

Among all tasks, low revolving-door and low-drawer operations had the highest sustained risk during the middle phase of job performance. In contrast, sliding-door and medium-height activities had lower and more stable risk trajectories. In addition, the confidence intervals were generally wider for low-level operations, suggesting greater inter-subject variability under these task conditions. These results suggest that cabinet height and operation type were associated with the temporal distribution of ergonomic risk, with low-level tasks imposing a more prolonged and variable postural burden on wheelchair users.

### 3.3. Joint Angle Distribution and Correlation Analysis

**Figure 7:**
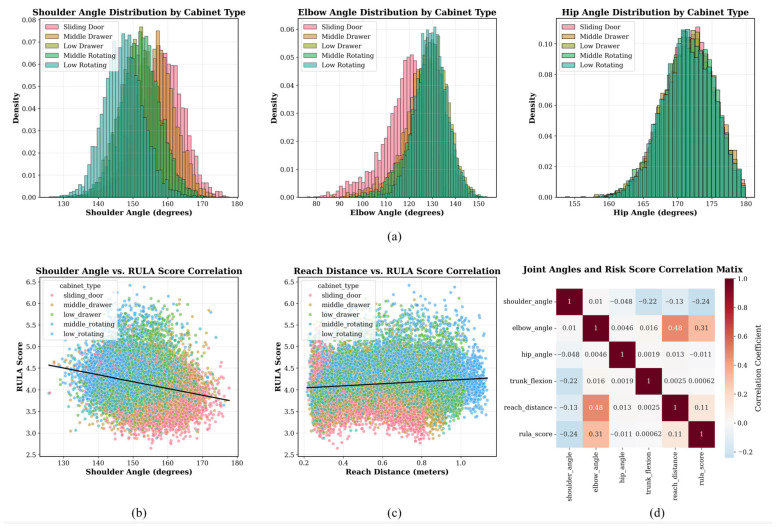
Joint angle distributions and risk correlations. (**a**) Distribution ranges of shoulder, elbow, and hip joint angles across cabinet-operation tasks. (**b**) Relationship between shoulder angle and ergonomic risk score, with fitted regression line. (**c**) Relationship between extension distance and ergonomic risk score. (**d**) Correlation matrix showing associations among kinematic variables and ergonomic risk scores. Among the examined variables, elbow angle showed the strongest correlation with ergonomic risk score (r = 0.31), whereas extension distance showed a weak positive correlation (r = 0.11).

Figure 7 comprehensively analyzes the distribution of joint angles in different cabinet operations and their correlation with ergonomic risk scores, revealing the biomechanical relationship.

When participants operated sliding doors or medium-height cabinets, shoulder angles were generally concentrated in the range of 140–160°. In contrast, low-level cabinet operations exhibited a greater shoulder angle dispersion, with more values between 130° and 140°. Elbow flexion peaked at 110–130° during low-drawer operations, indicating increased upper-limb postural effort.

Elbow angle was substantially correlated with ergonomic risk score (r = 0.31), indicating that higher-risk positions involved increased elbow flexion [48]. Hip joint angles varied between 165° and 175° across tasks, indicating that trunk-thigh configuration changed less than upper-limb posture throughout cabinet interaction. Extension distance revealed a weak positive relationship with ergonomic risk score (r = 0.11), implying that higher reaching demands enhanced ergonomic risk [52].

### 3.4. Risk Level Distribution Comparison

**Figure 8:**
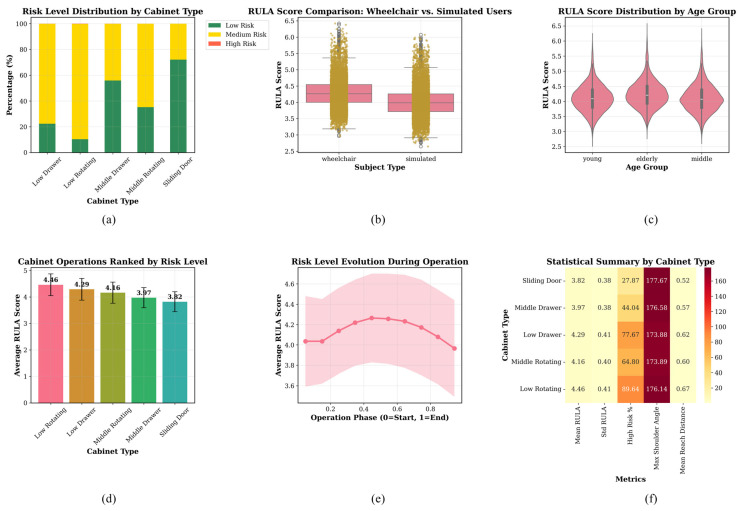
Comprehensive risk-level analysis. (**a**) Stacked bar chart showing the distribution of low-, medium-, and high-risk states across cabinet-operation tasks. (**b**) Boxplot comparison of risk scores between real wheelchair users and trained simulated operators. (**c**) Violin plot showing the distribution of risk scores across age groups. (**d**) Average risk scores for different cabinet-operation tasks, ranked from high to low, with error bars indicating inter-subject variability. (**e**) Mean risk evolution over the normalized operation phase. (**f**) Heatmap summarizing key indicators, including mean risk score, score standard deviation, high-risk proportion, maximum shoulder angle, and mean reach distance.

Figure 8 summarizes ergonomic risk distributions across cabinet-operation types, participant groups, and operation phases.

Among all tasks, low rotating-door operation had the highest average risk score (4.46), followed by low drawer (4.29), middle rotating door (4.16), middle drawer (3.97), and sliding door. A similar trend was seen in the fraction of high-risk exposure, with low rotating-door operation having the highest value (89.64%) and sliding-door operation having the lowest value (27.87%).

Comparing the two groups showed that the real wheelchair users had a higher average risk score than the trained simulated operators.

Across age groups, the elderly group showed a slightly higher central tendency and a somewhat broader score distribution than the young and middle-aged groups. In addition, the temporal trend in panel (e) indicates that risk increased from the beginning of the task, peaked during the middle phase of operation, and decreased toward the end.

The results show clear differences in ergonomic risk, depending on the type of cabinet operation. Specifically, low-level tasks, especially those involving rotating doors, create the most strain on posture.

### 3.5. Advanced Analytics and Model Performance

**Figure 9:**
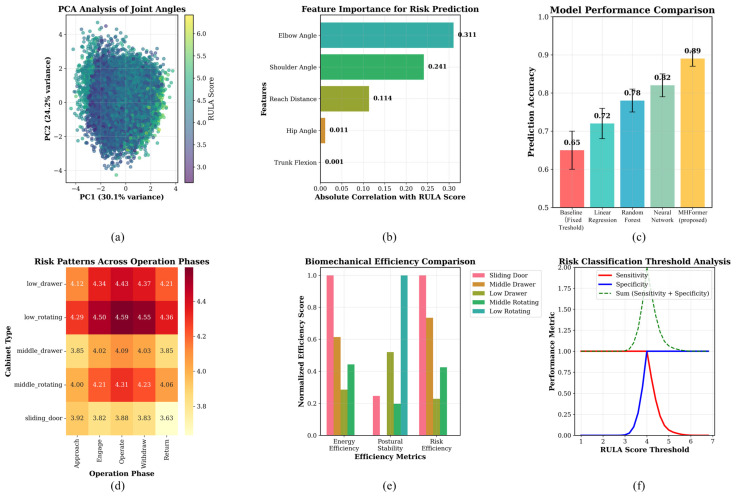
Advanced analytical results. (**a**) Principal component analysis (PCA) of joint-angle data, with each point color-coded by risk score. (**b**) Absolute correlations between selected kinematic variables and risk score. (**c**) Comparison of prediction accuracy across different models. (**d**) Heatmap showing risk-score variation across cabinet-operation types and task phases. (**e**) Comparison of normalized task-level efficiency metrics across cabinet-operation types. (**f**) Sensitivity, specificity, and their combined trend across different risk-classification thresholds.

Figure 9 shows additional joint-angle feature space, variable–risk relationships, model performance, task-stage risk patterns, normalized task-efficiency metrics, and threshold-based categorization behavior assessments.

The first two principal components explained 54.3% of the variation, with PC1 accounting for 30.1% and PC2 for 24.2%. Risk ratings did not form clearly separated clusters in the smaller two-dimensional feature space, showing that ergonomic risk varied continuously across posture configurations.

Elbow angle had the highest association with risk score (|r| = 0.311), followed by shoulder angle (0.241) and reach distance (0.114). In contrast, hip angle (|r| = 0.011) and trunk flexion (|r| = 0.001) showed only little correlation.

The MHFormer-based strategy outperformed the fixed-threshold baseline (0.65), linear regression (0.72), random forest (0.78), and neural network (0.82) in model comparison. Panel (d)’s heatmap demonstrated that low rotating-door and low-drawer operations had higher middle task phase risk scores than other cabinet types.

Panel (e) indicated that cabinet shape and operation mode affected biomechanical performance in various dimensions by affecting normalized task-level efficiency measures. Panel (f) showed that a risk-score threshold of 4.0 was best for binary high-risk classification in terms of sensitivity and specificity.

These studies confirmed the suggested framework by exhibiting a continuous risk structure in posture feature space, elbow angle as the most strongly linked variable, and higher classification performance.

### 3.6. Design Implications and Intervention Recommendations

In this study, “high-risk exposure” is defined based on the discretized RULA risk levels, where scores of 5–6 correspond to high risk and score 7 corresponds to very high risk. The reported exposure proportion is calculated as the ratio of frames classified into these categories to the total number of frames within each task sequence.

**Figure 10:**
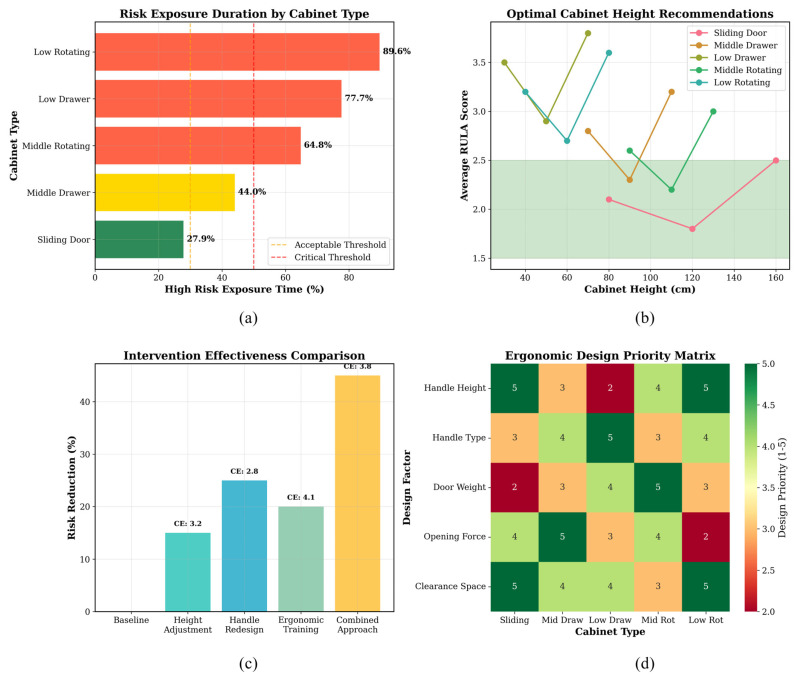
Design and intervention implications. (**a**) High-risk exposure duration across cabinet-operation types, with dashed lines indicating recommended interpretation thresholds for acceptable (<30%) and critical (>50%) exposure levels. (**b**) Simulation-based recommendation of cabinet or handle height in relation to average risk score across different cabinet-operation types. (**c**) Comparison of estimated risk reduction under different intervention strategies. (**d**) Design-priority matrix summarizing the relative importance of key ergonomic factors for different cabinet types.

Based on the observed distribution of high-risk exposure time, cabinet operations with exposure above 50%—such as low rotating-door (89.6%) and low drawer (77.7%) tasks—can be regarded as high-priority targets for ergonomic redesign. In contrast, sliding-door operation showed a high-risk exposure proportion of 27.9%, which remained below the proposed acceptable threshold. The simulation-based height analysis suggested that, for most cabinet-use scenarios, a handle or operating height of approximately 90–110 cm was associated with lower average risk scores. In the intervention comparison, the combined approach achieved the largest estimated risk reduction (45%), exceeding the effects of height adjustment alone (15%), handle redesign alone (25%), and ergonomic training alone (20%). The design-priority matrix further indicated that handle height and clearance space were consistently ranked as dominant factors across cabinet types, whereas the relative importance of door weight, opening force, and handle type varied depending on the cabinet configuration. Overall, these findings provide practical guidance for prioritizing ergonomic redesign strategies for wheelchair-accessible cabinet systems. The significance of the design and intervention is illustrated in Figure 10.

### 3.7. Benchmark Performance Evaluation

**Figure 11:**
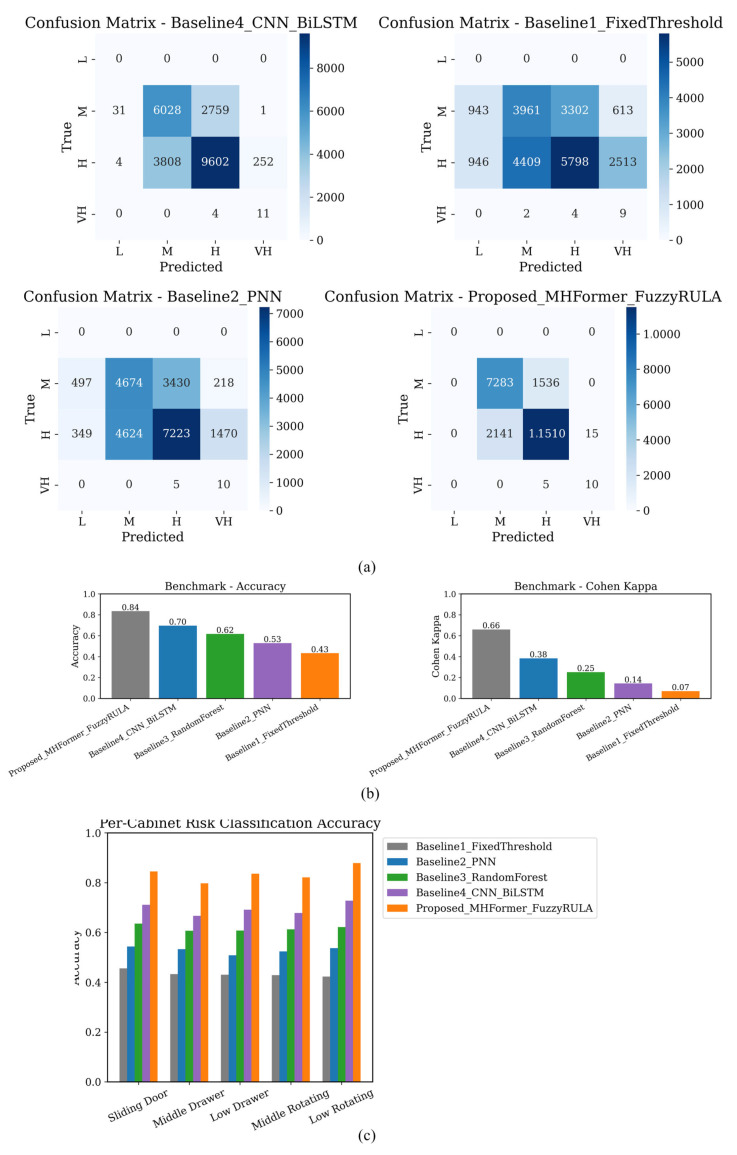
Benchmark performance comparison. (**a**) Confusion matrices for four representative methods, showing classification performance across four risk levels (L: low, M: medium, H: high, VH: very high). (**b**) Overall benchmark comparison in terms of accuracy and Cohen’s kappa for all five methods. (**c**) Per-cabinet classification accuracy across five cabinet-operation types.

Figure 11 presents a benchmark comparison between the proposed method and representative baseline approaches for risk-level classification.

To evaluate classification performance, the continuous ergonomic risk scores generated by the proposed fuzzy RULA model were discretized into four risk levels according to standard RULA criteria, namely low risk (score 1–2), medium risk (score 3–4), high risk (score 5–6), and very high risk (score 7). The reference labels were defined using the same categorization scheme. The classification accuracy was then computed by comparing the predicted risk categories, obtained through discretization of the continuous scores, with the corresponding reference labels. Therefore, the reported accuracy reflects the proportion of correctly classified samples across these four risk levels.

As shown in Figure 7b, the proposed MHFormer + Fuzzy RULA method achieved the best overall performance, with an accuracy of 0.84 and a Cohen’s kappa of 0.66. These values were higher than those of CNN-BiLSTM (0.70, κ = 0.38), Random Forest (0.62, κ = 0.25), PNN (0.53, κ = 0.14), and the fixed-threshold baseline (0.43, κ = 0.07).

The confusion matrices in Figure 7a demonstrate that the suggested method produced a stronger diagonal distribution than the representative baseline methods, indicating better agreement between anticipated and reference risk levels. In particular, the suggested model reduced misclassification within the main medium- and high-risk categories, which accounted for the majority of samples in the dataset.

Figure 7c demonstrates that the suggested technique maintained the highest classification accuracy across all cabinet operating modes. Its performance advantage was most noticeable in the more demanding low-level cabinet jobs, but it also regularly outperformed the baseline approaches in medium-level and sliding-door procedures. Overall, the results show that the proposed strategy gave the most robust and accurate risk classification among the approaches tested.

## 4. Discussion

This study examined an automated ergonomic assessment framework for wheelchair users during cabinet interaction by combining monocular vision-based 3D pose estimation with fuzzy RULA scoring. Taken together, the results suggest that this framework can capture posture-related risk in a way that is more sensitive to dynamic task demands than conventional static or threshold-based approaches.

The complementary roles of the three primary system components may explain this. Recent studies in intelligent prognostics have demonstrated that trustworthy modeling frameworks can effectively improve prediction reliability under limited data conditions, particularly in dynamic systems [53]. MHFormer was created to model several feasible posture hypotheses over time to solve monocular 3D pose reconstruction uncertainty [16]. This is important since cabinet operation often requires seated reaching, partial self-occlusion, and wheelchair interference. Recent work on automated RULA calculation has shown that motion-based ergonomic scoring may be extended beyond manual observation, making continuous posture evaluation more practical. Our results support that direction, but they also suggest that fuzzy logic reduces the rigidity of discrete scoring, especially for tasks where risk fluctuates gradually [14,17].

The improvement in joint-angle estimation error relative to the Kinect v2 baseline also supports the practical value of the proposed framework. More importantly, the temporal results show that ergonomic risk is not evenly distributed throughout cabinet operation. Instead, risk rises during reaching and task engagement, peaks in the middle phase, and then declines as the user returns toward a more neutral posture. This pattern matters because it indicates that the most demanding part of the task is concentrated in a relatively short interval rather than spread uniformly across the whole movement. From a design or feedback perspective, that middle phase may be the most meaningful target for intervention.

The joint-angle analysis provides a more specific explanation of where this burden may come from. Among the variables examined, elbow angle showed the strongest association with the risk score, although the magnitude of that relationship was still moderate. In other words, elbow posture should not be treated as the sole determinant of ergonomic load, but it may serve as a useful practical indicator when screening cabinet operations for elevated risk. This interpretation is also consistent with the broader ergonomic literature, where upper-limb posture and repeated non-neutral arm configurations are often closely tied to task difficulty and musculoskeletal burden [51,52].

Another point that deserves attention is the difference between experienced wheelchair users and trained simulated participants. The simulated group was useful for controlled comparison, but the higher scores observed in real wheelchair users suggest that short-term simulation cannot fully reproduce long-term movement strategies, wheelchair fit, or compensatory habits formed through daily use. In practical terms, this means that studies relying only on simulated participants may underestimate the postural demand associated with real household tasks.

The differences among cabinet types were also clear. Low rotating-door and low-drawer tasks produced both higher risk scores and longer high-risk exposure than the other operations, while sliding-door tasks showed the lowest overall burden. This result fits reasonably well with previous work on kitchen and furniture ergonomics, which has emphasized that storage height, access depth, and cabinet configuration can strongly influence reachability and comfort, especially for older adults and users with mobility limitations [54]. Our data extend those observations by showing not only that some cabinet configurations are harder to use, but also when during the movement that extra burden is most likely to occur.

The design-oriented analyses point in the same direction. A handle or operating height around 90–110 cm was associated with lower average risk in many of the tested scenarios, but height alone was not enough to explain overall usability. Opening force, handle form, and operating clearance also influenced the final posture pattern. This is one reason the combined intervention scenario performed better than any single adjustment alone. Rather than treating accessibility as a matter of one-dimensional reach range, the present findings suggest that cabinet design should be approached as a multi-factor ergonomic problem. That view is also compatible with work in inclusive and universal kitchen design, where clearance, usability, and task comfort are considered together rather than in isolation [55].

More broadly, the present framework adds something that basic accessibility checks often miss. A cabinet may be technically reachable and still impose awkward, sustained, or repeated postures during use. By quantifying not only score level but also exposure time and task-specific variation, the current approach provides a more detailed description of ergonomic demand. For that reason, it may be useful as a supplementary evaluation tool when barrier-free furniture design is being assessed or refined.

The method also has some practical promise beyond offline analysis. In addition, recent advances in few-shot learning and meta-learning have shown strong potential for improving model generalization under limited or variable data conditions [56], which may further enhance the applicability of the proposed framework in real-world assistive environments. Because it relies on monocular RGB input instead of specialized motion-capture equipment, it may be easier to integrate into smart-home or assistive-monitoring settings. With further validation, such systems could potentially support real-time feedback during daily activities or identify repeated patterns of postural exposure that justify environmental adjustment.

## 5. Limitations and Future Work

This study should still be interpreted within the scope of its design. The experiments were conducted under controlled laboratory conditions and focused on five representative cabinet-operation tasks. That setup was useful for building a clear comparison framework, but real homes are more variable. Furniture layout, available maneuvering space, lighting, and multitasking demands may all affect how wheelchair users actually move during everyday cabinet use.

A second point is that the current framework is built on monocular RGB input. This makes the method relatively practical and easier to deploy, but it also means that depth reconstruction remains less precise than in professional multi-camera or motion-capture systems. In addition, although both experienced wheelchair users and trained simulated participants were included, the overall sample size was still limited, so the results should be treated as encouraging rather than final.

Future work can therefore move in three main directions. First, the framework should be tested in more realistic home environments and across a wider range of furniture interactions. Second, it would be valuable to develop more personalized risk models by incorporating anthropometric variation, wheelchair configuration, and individual movement habits. Third, longer-term studies are needed to examine whether repeated exposure to the kinds of postural demands identified here is associated with pain, fatigue, or other musculoskeletal outcomes over time.

In addition, although Kinect v2 was adopted in this study as a representative low-cost baseline for ergonomic motion capture, it is acknowledged that newer sensing technologies, such as Azure Kinect and recent deep learning-based pose estimation methods, may offer improved accuracy and robustness. Future work will include comparisons with these advanced approaches to further validate and generalize the proposed framework.

Overall, the present findings provide a useful basis for subsequent research and system refinement. With further validation and broader application, the proposed framework may offer practical value for ergonomic evaluation, accessible furniture design, and future assistive or smart-home applications.

## 6. Conclusions

This work proposes a thorough approach for automated wheelchair user ergonomic risk assessment during cabinet operation, expanding barrier-free design assessment beyond accessibility evaluation to quantitative ergonomic analysis. The suggested method advances earlier approaches by including YOLOv11-based target detection, Multi-Hypothesis Transformer position estimation, and fuzzy logic-enhanced RULA evaluation. It also serves as a practical reference for barrier-free furniture design and ergonomic optimization.

The main contributions of this study can be summarized in three aspects. First, the cascaded YOLOv11 + MHFormer architecture substantially improved joint-angle estimation accuracy compared with the depth-sensor baseline, reducing the average RMSE by about 60%. Second, the fuzzy RULA enhancement allowed ergonomic risk to be expressed in a more continuous way, which made it possible to better describe the gradual accumulation of postural stress during cabinet operation. Third, the overall system achieved 84% classification accuracy and a Cohen’s kappa value of 0.66, showing better agreement with expert evaluation than the baseline methods considered in this study [16,50].

Based on the analysis of 30 participants across five cabinet-operation tasks, this study identified clear differences in ergonomic burden among cabinet types and extracted several practically meaningful risk characteristics. Among the examined kinematic variables, elbow angle showed the strongest association with risk score (r = 0.31), suggesting that it may be useful as a simplified indicator in posture-related risk screening. The temporal results further showed that risk was not evenly distributed across the task, but tended to peak during the middle stage of operation, providing useful information for intervention timing and task redesign. In addition, the design-priority analysis and height-related results offer practical reference for barrier-free furniture design, while the combined intervention analysis suggested an estimated risk reduction of up to 45%.

The risk exposure results also showed that some cabinet configurations imposed substantially greater burden than others.

The low revolving door held people in a high-risk posture for 89.6% of the time, indicating that ergonomic improvements should be prioritized for this design. Lower-risk tasks, such as operating a sliding door, may be more suitable for daily use. The contrast between experienced wheelchair users and simulated participants underscores the need of integrating real users in ergonomic validation, as laboratory simulation may not adequately represent long-term adaption procedures and postural demands [1,52]. According to the findings of this study, cabinet accessibility should be evaluated using reachability, postural stress, exposure length, and task-specific movement demand metrics. The framework describes how computer vision and ergonomic evaluation can be utilized in barrier-free design research to better understand how different cabinet designs affect wheelchair users during actual use. This method could be effective in accessible housing evaluation, smart-home environments, and long-term ergonomic monitoring with validation.

## Figures and Tables

**Table 1:** Representative fuzzy RULA inference rules for ergonomic risk assessment.

Rule ID	Shoulder Abduction	Elbow Flexion	Trunk Inclination	Risk Level
R1	Low	Neutral	Upright	Low
R2	Medium	Neutral	Slight Forward	Medium
R3	High	Moderate	Slight Forward	High
R4	High	Extreme	Bent	Very High
R5	Medium	Moderate	Bent	High
R6	Low	Moderate	Upright	Medium
R7	High	Neutral	Twisted	High
R8	Very High	Extreme	Twisted	Very High

**Table 2:** Configuration of loss function hyperparameters.

Hyperparameter	Value	Functional Role in Cabinet Operation Scenarios
Localization Weight ($\lambda_{1}$)	7.5	High Priority: Severe punishment for boundary box errors. In high occlusion tasks (such as arm crossing body when operating a medium height revolving door), this mechanism is very important to maintain the user’s attention.
Keypoint Weight ($\lambda_{2}$)	0.5	Refinement: after the global bounding box is correctly established, fine tune the internal joint position (shoulder joint, elbow joint).

**Table 3:** Training configuration and hyperparameters of YOLOv11.

Parameter	Value	Description
Input resolution	640 × 640	Input image size for YOLOv11
Batch size	16	Number of samples per batch
Learning rate	0.01	Initial learning rate
Optimizer	SGD	Optimization algorithm
Momentum	0.937	SGD momentum
Weight decay	0.0005	Regularization parameter
Epochs	300	Total training epochs

**Table 4:** The relevant information of the participants in the dataset construction process.

Group	Young Adults (≤44 Years)	Middle-Aged Adults (45–59 Years)	Older Adults (≥60 Years)	Total
Experimental Group	4	5	5	14
Simulation Group	10	4	2	16

**Table 5:** The relevant information of cabinet furniture in the dataset construction process.

Cabinet Type	Handle Height from Floor	Opening Direction	Schematic Diagram
Sliding Cabinet Door	Any height	Horizontal sliding	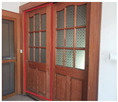
Mid-Height Drawer	70 cm	Push-pull	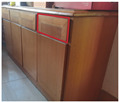
Low-Height Drawer	16 cm	Push-pull	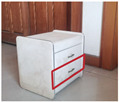
Mid-Height Hinged Cabinet Door	120 cm	Clockwise rotation	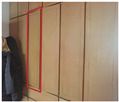
		Counterclockwise rotation	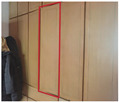
Low-Height Hinged Cabinet Door	45 cm	Clockwise rotation	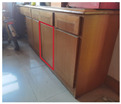
		Counterclockwise rotation	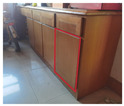

**Table 6:** Dataset construction task workflow.

Task Order	Task Description	Schematic Diagram
1	The experimenter starts the video recording equipment to begin the experimental procedure.	-
2	The participant maneuvers the wheelchair into the experimental area and ensures the wheelchair is stably positioned at the designated location.	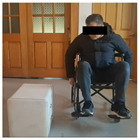
3	The participant opens the corresponding cabinet door, demonstrating the door-opening action in actual operation.	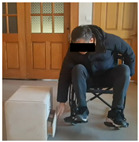
4	The participant retrieves a preset item from the cabinet, simulating a daily object retrieval scenario. The reaching and grasping movements are recorded.	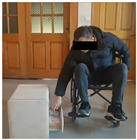
5	After retrieving the item, the participant closes the cabinet door, completing the entire retrieval process.	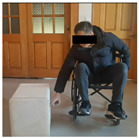
6	The participant returns their hand to the wheelchair’s handrim, indicating the completion of the operation task.	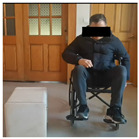
7	The experimenter stops the video recording, concluding the experimental session.	-

**Table 7:** Joint angle estimation RMSE comparison (degrees).

Posture Movement	Kinect v2 RMSE	Our Method RMSE
Raising Arms	12.5°	7.1°
Holding Objects with Both Hands	20.5°	7.9°
Extending Arms	18.3°	7.3°
Neck Flexion	19.6°	6.8°
Forward Trunk Flexion	22.1°	8.2°
Average	18.6°	7.5°

## Data Availability

The source code of this study is available upon request. However, the videos from furniture factories used in data collection are not publicly accessible, as this is necessary to protect the privacy and personal information of the workers involved in this research.

## Abbreviations

WMSDs	Work-related musculoskeletal disorders
MSDs	Musculoskeletal diseases
RULA	Rapid upper limb assessment
REBA	Rapid entire body assessment
WUSPI	Wheelchair user’s shoulder pain index
PA	Posture angle
MHFormer	Multi-hypothesis transformer
MHG	Multiple hypothesis generation
SHR	Self hypothesis refinement
CHI	Cross-hypothesis interaction
GCNs	Graph convolution networks
YOLO	You only look once
RMSE	Root mean square error
MAE	Mean absolute error
MPJPE	Mean joint position error
PCA	Principal component analysis
CNN	Convolutional neural network
BiLSTM	Bidirectional long short-term memory
FSA-HFFormer	Frequency-spectral adaptive hierarchical fusion transformer
LSP-YOLO	Lightweight single-stage pose YOLO
